# Pathophysiology of the Behçet's Disease

**DOI:** 10.1155/2012/493015

**Published:** 2011-10-01

**Authors:** Ümit Türsen

**Affiliations:** Department of Dermatology, School of Medicine, Mersin University, Zeytinlibahce, 33079 Mersin, Turkey

## Abstract

Behçet's disease (BD) is a multisystemic disease of unknown etiology characterized by chronic relapsing oral-genital ulcers and uveitis. Multiple systemic associations including articular, gastrointestinal, cardiopulmonary, neurologic, and vascular involvement are also observed in BD. Although the etiopathogenesis of the disease remains unknown, increased neutrophil functions such as chemotaxis, phagocytosis, and excessive production of reactive oxygen species (ROS), including superoxide anion, which may be responsible for oxidative tissue damage seen in BD, and also immunological alterations, T lymphocyte abnormalities in both subpopulation and function have been considered to be correlated with the etiopathogenesis of BD. There is some clinical evidence suggesting that emotional stress and hormonal alterations can influence the course and disease activity of BD.

## 1. Introduction

Behçet's disease (BD) is a recurrent systemic inflammatory disorder characterized by four major symptoms consisting of oral aphthous ulcers, ocular lesions, skin lesions, and genital ulcerations. Although many studies have been conducted on the etiopathogenesis of the disease, exact mechanisms have not yet been fully understood [1]. Multiple systemic associations including articular, gastrointestinal, cardiopulmonary, neurologic, and vascular involvement are also observed in BD [1, 2]. Although the etiopathogenesis of the disease remains unknown, increased neutrophil functions such as chemotaxis, phagocytosis, and excessive production of reactive oxygen species (ROS), including superoxide anion, which may be responsible for oxidative tissue damage seen in BD, and also immunological alterations, T lymphocyte abnormalities in both subpopulation and function have been considered to be correlated with the etiopathogenesis of BD. It was postulated that Behçet's disease is an autoimmune disease. Systemic manifestations such as articular, gastrointestinal, and neurologic manifestations are not associations with the disease. They are different involvements due to the disease [3, 4]. There is also some clinical evidence suggesting that emotional stress and hormonal alterations can influence the course and disease activity of BD [5–7].

## 2. Immune System Dysregulations

**Figure 1:**
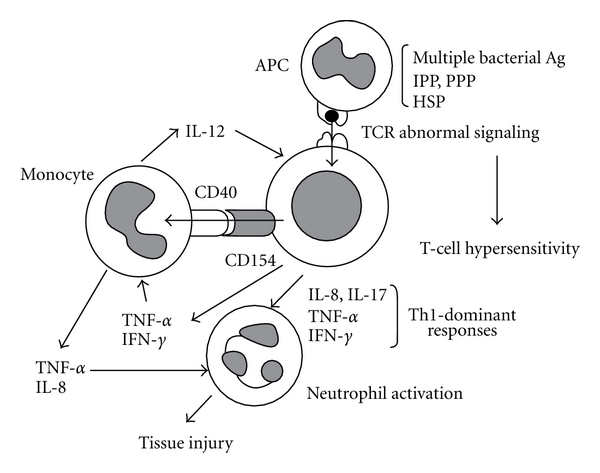
Proposed model of the pathogenesis in Behçet's disease. Ag: antigen; APC: antigen-presenting cells; HSP: heat shock protein; IFN: interferon; IL: interleukin; IPP: isoprenyl pyrophosphate; PPP: prenyl pyrophosphate; TCR: T-cell receptor; Th1: T-helper cells type 1; TNF-*α*: tumor necrosis factor *α*.

The immunopathogenesis that is currently postulated is shown in Figure 1. Primarily, hypersensitivity of T cells (*αβ*T cells and *γδ*-T cells) to multiple antigens appears to play a critical role in the pathogenesis. The activation of monocytes subsequent to T-cell activation through CD40–CD154 interactions as well as a variety of T-cell-derived cytokines (IFN-*γ* and TNF-*α*) may result in the production of IL-12, which leads to the shift to Th1 responses. In consequence of abnormal T-cell activation, neutrophil activation may be triggered by cytokines such as IL-8, IL-17, IFN-*γ*, and TNF-*α*. Whereas the roles of costimulation molecules have not been fully explored in Behçet's disease, the presence of anti-CTLA-4 antibody has been reported in a fraction of Behçet's disease patients. Although the presence of this antibody might be possibly involved in abnormal T-cell responses, the antibody might be produced only as a secondary phenomenon of recurrent T-cell activation in Behçet's disease [7, 8].

## 3. Cellular and Humoral Immunology

Although cellular activity is increased in the peripheral blood of BD patients, reports of neonatal BD cases in the children of affected women also suggest a role for humoral factors. Indeed, various cytokine profiles and elevated lymphocyte populations have been demonstrated in BD with an imbalance between Th1- and Th2-phenotype lymphocyte components of the immune response. However, as T lymphocytes are particularly responsive to antigens of viral or bacterial pathogens, the skew is therefore suggested toward Th1-phenotype lymphocyte response followed by an infiltration into the affected regions [7, 8].

### 3.1. Th1-Phenotype Lymphocytes

Th1-phenotype lymphocytes that produce proinflammatory mediators called cytokines, such as IL-2, IL-6, IL-8, IL-12, IL-18, TNF-*α*, and IFN-*γ* are increased in patients with BD [7].

### 3.2. Th2-Phenotype Lymphocytes

Th2 cytokines have responses precisely opposite to those of reactions elicited by Th1. The results regarding the Th2-phenotype lymphocytes and cytokines are controversial. Some studies have shown decreased levels of CD8 T lymphocytes, IL-4, and IL-10, whereas some others demonstrated increased CD8 T-lymphocyte populations as well as increased serum concentrations of IL-4, IL-6, IL-10, and IL-13, indicating a reduced circulating CD4/CD8 ratio [7].

### 3.3. Immunoglobulins, Immune Complexes, and Anticardiolipins

Enhanced cell-mediated cytotoxicity with demonstrated circulating immune complex response (usually antigen-antibody complexes) against oral mucosal antigens, especially during an exacerbation period, supports the presence of both Th1 and Th2 types of immune reaction in BD. These immune complexes may be priming factors that trigger the disease with a recruitment of some immune cells to the site of inflammation that are present in the sera of more than one-half of BD patients [7].

### 3.4. Neutrophils, Monocytes, and Complements

There is a generalized derangement of the lymphocyte and neutrophil populations during the course of BD, which is characterized by elevated peripheral white blood cell count, activated monocytes, increased neutrophil motility with infiltration into the cutaneous and ocular lesions, and increased circulating proteins such as C3, C4, C5, IgA, Haptoglobin, and orosomucoid [9]. Active monocytes produce a number of proinflammatory cytokines, such as IL-1, IL-6, IL-8, TNF-*α*, and granulocyte-macrophage colony stimulating factor (GM-CSF), and these cytokines contribute to neutrophil activation by their augmented interactions with endothelial cells, causing tissue damage possibly by priming neutrophils [10]. Indeed, various hyperfunctions of neutrophils in peripheral blood such as chemotaxis, active oxygen production, and phagocytosis with infiltration into the lesion sites have all been implicated during the course of active BD [11]. Moreover, leukocyte adhesion molecules such as P and L selectins, Mac-1, and CD4 are expressed on peripheral leukocytes and participate in the cascade of leukocyte chemotaxis and adhesion, indicating the presence of immune system activation in BD [12]. Plasma myeloperoxidase (MPO) activity, representing neutrophil activation, and biomarkers of oxidative stress reflecting protein oxidation, such as the levels of advanced oxidation protein products (AOPPs), have been found to be increased in BD patients, especially in active disease [13]. This suggests again the activated neutrophils in the etiopathogenesis of BD.

### 3.5. Heat Shock Proteins in BD

Heat shock protein (HSP) is a small, ubiquitous stress-related streptococcal antigen that can be induced by infections, trauma, heat, UV-B, hypoxia, cold, and cytotoxic prostaglandins [14]. Anti-HSP-65 antibodies cross-reactive with oral mucosal homogenates and oral streptococci have been reported in BD. T-cell antigen receptor (TCR) *γδ*-T lymphocytes are important in first-line defense that allows T cells to recognize a wide range of antigens as well as in regulation of Th1-Th2 responses, and HSP itself activates these subsets of cells known as the CD4 and *γδ*-T lymphocytes that do not require peptide-MHC I or II association for stimulation. Likewise, microbial HSP-65-derived peptides and their mammalian counterpart HSP-60, both of which cause autoimmunity with 60% of sequence homology, have recently been demonstrated to stimulate lymphoproliferative response in BD patients [14, 15].

Recent developments in the innate immune system with the description of toll-like receptors (TLRs) and HSP-60 as a ligand for TLR-2 and TLR-4 suggest also the role of HSP-60 as an endogenous “danger” signal to the immune system with rapid inflammatory cytokine release and the enhancement of adaptive Th1-type responses [16]. Although contradictory results have been reported for peripheral blood TCR *γδ*-T lymphocyte counts, HSPs have pathophysiologically been suggested in BD because increased T- and B-cell responses against these autoantigens are observed in different ethnic populations in BD [17]. Therefore, these cells as well as HSPs may participate during the course of ocular and mucocutaneous BD because of the following: (1) antibodies against bacterial or human HSP-60/65 are capable of cross-reacting with retinal antigens; (2) exposure to exogenous or endogenous HSPs results in the proliferation of peripheral T lymphocytes of ocular BD patients directed against both the target and retinal antigens; (3) lesional skin of BD contains increased numbers of both HSP-60 and TCR *γδ*-T lymphocytes; (4) *γδ*-T lymphocyte is present in tissue specimens whereas HSP-65 expression is abundantly upregulated in epidermal regions of active skin lesions such as erythema nodosum and mucocutaneous lesions of patients with BD; finally (5) elevated HSPs upregulate the expression of the MICA locus in BD patients lying adjacent to the HLA-B5101 locus [18, 19]. Moreover, increased TCR-*γδ*-T lymphocytes is normally cultured from inflamed vitreous and have been detected in cerebrospinal fluid (CSF) and bronchoalveolar lavage of patients with active BD. Furthermore, subcutaneous inoculation or oral administration of HSP-derived peptide induces experimental uveitis [20]. Taken together, BD seems to result from heightened responsiveness to bacterial antigens in genetically susceptible hosts. In other words, streptococcal HSP epitopes may reach the submucosa of the mouth ulcers and elicit an inflammatory reaction through upregulated HSP expression by minor injuries, which stimulate self-HSP-60 reactive clones, suggesting different local HSP responsive T-lymphocyte repertoire from that of peripheral blood. This, in turn, may serve as a local antigen with augmentation of inflammatory reaction.

### 3.6. Oxidative Stress, Antioxidative Defense, and Trace Elements in BD

In spite of unknown aetiology, it is now accepted that reactive oxygen species produced by neutrophils may be related to the pathogenesis of BD. Not only increased malondialdehyde and superoxide dismutase levels but also decreased glutathione peroxidase activities in erythrocytes were observed in patients with BD [21]. Toxic compounds such as pesticides and insecticides have been incriminated in BD. Recently, Aynacioglu et al. showed that N-acetyltransferase 2*5B allele was slightly higher in patients with BD. This enzyme contributes to drug and toxic compound metabolism [22]. Excessive superoxide anion (O_2_^−^) production and raised ADA activity (a marker of activated neutrophil function, chemotaxis, and phagocytosis), as well as hydrogen peroxide- (H_2_O_2_-) induced hydroxyl radical (OH) and malondialdehyde productions have been demonstrated in BD patients, suggesting neutrophil-mediated immunity and increased amount of reactive oxygen species (ROS) production, especially in the exacerbation period [23]. Indeed, neutrophils of active BD patients are much more vulnerable to oxidative injury than those from inactive patients. On the other hand, endogenous free radical scavenging enzymes, such as superoxide dismutase, glutathione peroxidase, and catalase, have been found to be decreased in patients with BD [24]. This clearly results in insufficient disposal of O_2_^−^ and H_2_O_2_ and, therefore, limited enzymatic adaptation to ROS with circulating prooxidants in such patients [25]. As activated T lymphocytes cause neutrophil hyperfunction with overproduction of NO, O_2_^−^, H_2_O_2_, OH, and singlet oxygen (_1_O_2_) that is one of main ROS-generation system in BD patients, the increased production of ADA and LPO system confirms aggravated lymphocyte function and ROS production, suggesting a possible new and simple biochemical activity marker for ADA in BD. Therefore, the interplay between ADA, O_2_^−^, NO, and peroxynitrite anion in the vascular wall of BD patients is likely to cause antioxidant enzyme depletion, oxidative LPO and, therefore, deteriorated oxidant/antioxidant equilibrium, creating a condition known as oxidative stress [26]. Trace elements function as cofactors to antioxidant enzymes. Erythrocyte selenium, plasma iron, manganese, and zinc levels are decreased, whereas plasma copper, erythrocyte zinc, and manganese levels are elevated in patients with BD. In addition, the plasma concentrations of powerful nonenzymatic antioxidants such as vitamins A, C, E, and *β*-carotene are lower in such patients [27, 28].

### 3.7. Endothelial Cells, Nitric Oxide, and Related New Inflammatory Molecules

Behçet disease is characterized by vasculitis and endothelial cell dysfunction. Nitric oxide (NO), endothelium-derived relaxing factor, is a free oxygen radical that is produced by endothelial cells upon stimulation by immunologic, infectious, and inflammatory stimuli, such as cytokines, INF-*γ*, lipopolysaccharides, and endotoxin [29]. It is an important mediator of uveal inflammation, and NO synthase activity has experimentally been demonstrated in the uveal tissue [30]. Recently, evidence is accumulating for the role of NO during the course of BD. Serum nitrite and nitrate concentrations as an indicator of recent NO production have been found to be decreased in BD patients [31], and three recent studies have shown that Glu-Asp298 polymorphisms of endothelial NO synthase gene are associated with BD susceptibility [32]. In another study, serum, erythrocyte, and synovial NO have been demonstrated in BD [33]. NO concentrations are increased in BD patients and associated with disease activity. Similar results have been obtained by various investigators supporting this finding [34]. In addition, aqueous humor NO levels have been reported to be increased in uveitic BD patients [35]. Increased serum levels of NO may be explained by various molecules that have recently been implicated during the course of BD. First, homocysteine, which is found to be elevated in BD [36], enhances NO synthesis from endothelial cells, induces the expression of chemoattractants by oxygen free radicals, and is the potent inducer for IL-6, IL-8, and TNF-*α* [37]. Because increased levels of proinflammatory cytokines by endothelial cells, neutrophils, and macrophages have well been established during the course of BD [38], homocysteine- and cytokine-induced overproduction of NO by immunocompetent cells may pathophysiologically be related with BD and uveitis due to NO-generating cells such as the endothelium, neutrophils, and macrophages, resulting in oxidative stress with self-propagating LPO in such patients [39]. Elevated NO levels, in turn, may compensate these effects of homocysteine by its adhesion-inhibitory properties. Second, another endothelium-specific cytokine, vascular endothelial growth factor, is produced by macrophages, activated human neutrophils, monocytes, and vascular endothelial cells and potently stimulates angiogenesis, endothelium-dependent vasodilatation, and NO production by its receptors located on the systemic and retinal vascular endothelial cells [40]. Indeed, inflammation and proinflammatory cytokines induce VEGF expression and VEGF itself upregulates NO synthase expression in endothelial cells, inducing large amount of NO production and leukocyte mobilization [41]. Because serum VEGF levels have been found to be increased in BD patients and correlated with ocular disease with demonstrated VEGF gene polymorphisms [42], VEGF may therefore have contributed to the elevated NO levels along with an additional risk factor for the development of retinal vaso-occlusive disease and neovascularization, resulting in poor visual outcome in such patients. Finally, leptin, a product of the recently cloned *ob* gene, is expressed in human vasculature and endothelial cells and plays a crucial role during inflammation, and impaired endothelial function reverses after leptin replacement [43]. TNF increases serum leptin levels in human, and leptin itself directly enhances the release of NO from endothelial cells, suggesting an autocrine or paracrine modulator role [44]. Because serum leptin levels have been demonstrated to be higher in acute-phase response as well as in BD patients [45], the pathophysiological significance of homocysteine-cytokine-VEGF-leptin-NO cascade should further be investigated in detail during the course of BD. 

## 4. The Role of Stress Factors and Stress Hormones in BD

### 4.1. Hypothalamo-Pituitary Adrenal Axis in Behçet's Disease

Dysfunction of the hypophysis gland in both humans and animals was shown to be associated with several autoimmune diseases [46, 47]. As in other chronic autoimmune diseases, there may be some changes in cortisol levels due to adrenal tiredness associated with long-term stress in Behçet's disease. Colak et al. observed that cortisol values in the 60th minute were significantly lower in Behçet's disease patients than in the control group after 1 *μ*g ACTH stimulation test. In this study, when peak cortisol responses to low-dose test were compared between patient and control groups, a significant decrease was found in the patient group. When peak cortisol responses to low-dose test and standard-dose test were compared in the patient group, peak cortisol responses to low-dose test were found significantly lower than those to standard-dose test. Comparison of under the curve cortisol responses between patient and control groups showed that under the curve value was significantly lower between 30th and 60th minutes in low-dose test in the patient group than in the control group. When percentage increase values of cortisol responses to low-dose test were compared between patient and control groups, the 60th minute value in the patient group was found significantly lower than that in the control group [48]. Autoimmune diseases develop when endocrine changes caused by various stresses like infection, together with some regulatory defects, affect autoreactive cells and exceed the critical threshold that leads to autoimmunity [49]. The most common cause of primary adrenal cortex failure is autoimmune adrenalitis that results in bilateral adrenal atrophy. Clinical signs of adrenal failure are not seen until at least 90% of the adrenocortical tissue is destroyed. The rate of the copresence of diseases having autoimmune events in their pathogenesis is high. Primary adrenal failure, which is an autoimmune disease, may accompany Behçet's disease, for the aetiopathogenesis of which autoimmunity is held responsible [50]. There are some studies assessing adrenal functions in the other autoimmune diseases including rheumatoid arthritis, systemic lupus erythematosus, and Sjögren's syndrome [51–55]. There is a partial dysfunction that can be revealed by LDT in Behçet's syndrome patients [48]. It was concluded that hypothalamo-pituitary adrenal axis was partially suppressed in Behçet's disease and that occult adrenal failure should be carefully considered in case of acute stress.

### 4.2. Sex Hormones in BD

While this paper has focused on the HPA axis and glucocorticoids and their role in susceptibility to inflammatory disease, estrogen is known to play an extremely important role in immune modulation and contributes to the approximately two- to tenfold higher ratio of most autoimmune diseases in females of all species [56]. Ovariectomy has been shown to reduce, while replacement of estrogen reconstitutes, this differential susceptibility to experimental inflammatory arthritis in rodents [57]. Furthermore, gender, menstrual cycle, and estrogen replacement therapy have all been shown to affect HPA axis and immune function in human studies [58].

### 4.3. Activation of Neutrophils by Testosterone in Behçet's Disease

To determine the putative role of testosterone on neutrophil activity exhibited by patients with BD, peripheral blood neutrophils were examined in vitro before and after treatment with testosterone. Yavuz et al. analysed peripheral blood neutrophils of 49 patients with BD, 33 patients with ankylosing spondylitis, 8 female patients with hirsutism, and 31 healthy individuals. They indicated that gender differences were striking not only in the mean oxidative burst response but also in the rate of apoptosis. Male BD patients manifested increased burst response before testosterone treatment compared with females. Consistent with oxidative burst results, baseline percentages of CD66b- and CD16-expressing cells were greater in male BD patients. A decreased apoptosis ratio was observed using PhiPhilux and PI staining in BD patients. This was especially significant in male compared to female BD patients. BD itself rather than the gender was found to be the most important predictor of this altered apoptosis ratio in BD determined by linear regression analysis [59]. These results suggested that a contribution of testosterone to the BD pathogenesis could not be ruled out. 

### 4.4. Prolactin (PRL) and Other Sex Hormones in BD

Prolactin acts as a neuroendocrine modulator of both skin epithelial growth and the skin immune system. Moreover, it was proposed that PRL forms a “PRL circuit” between the skin and the central nervous system [60]. This concept can now be readily integrated into current views on the multilevel neuroendocrine-immune communication along the “brain-skin axis” in health and disease [61]. Inspired by this hypothesis, PRL and PRL-receptors expression have now been demonstrated in several cutaneous cell populations, including keratinocytes, fibroblasts, sweat glands, and sebaceous glands. In addition, PRL and PRL-receptors expression has also been identified in the key cellular protagonists of the skin immune system [62]. 

The results regarding the PRL levels in BD are controversial. Some studies have shown increased levels of PRL, whereas some others demonstrated decreased and normal levels of PRL in BD [63–66]. Karakus et al. indicated that PRL levels of male patients having ocular involvement had lower levels of inactive group, while female patients having ocular involvement had lower levels of DHEA-S compared to the active group. They found that no other remarkable hormonal difference including FSH, LH, T3, T4, TSH was observed among Behçet's patients [67]. Gül et al. found that mean total testosterone levels of BD patients were significantly lower than those of healthy controls; however, patients with BD and increased 17-OH-progesteron levels only had normal total testosterone levels. And they also indicated more commonly 21-hydroxylase gene mutations and deficiency in BD [68]. Mat et al. found that androgen receptor density in scrotal skin of BD was normal [69]. In some studies, it has been suggested that there is a correlation between high serum prolactin levels and activation of certain autoimmune diseases. Hyperprolactinemia, which has the potential to exacerbate autoimmunity, may coexist with BD [70]. For many years, bromocriptine, a D1 and D2 dopamine receptor agonist, has been the standard medicine for hyperprolactinemic patients. That there was a beneficial effect of low-dose cyclosporine plus bromocriptine combination therapy on autoimmune human uveitis may indicate the role of prolactin in the pathogenesis of BD [70]. 

### 4.5. Neuropeptides in Behçet's Disease

Recently, the association between stressful life events and various dermatologic diseases is explained by the concept of neuro-immune-cutaneous system [71]. It was reported that neuropeptides, especially substance P (SP) and calcitonin-gene-related peptide (CGRP), and neurotrophins such as nerve growth factor (NGF) affect the pathogenesis of skin disorders like atopic dermatitis and psoriasis vulgaris [72, 73]. Taking into account inflammatory responses that occur during emotional stress in BD patients, it seems likely that neurogenic mediators may be associated with BD. Jang et al. observed that strong immunoreactivity of SP and NGF was seen in the epidermis, panniculitis lesion, and vasculitis lesion of BD patients. However, CGRP levels were decreased in BD patients [74]. Aki et al. noted that SP in the active BD period was significantly higher than controls whereas that of inactive BD was not. SP in active BD was significantly higher than in inactive BD. And also they found that CGRP during both active and inactive periods of BD was significantly higher than controls, and CGRP in active BD was significantly higher than in inactive BD. They think that the increased SP and CGRP found in active BD may be associated with BD pathogenesis through the increase in expression of cellular adhesion molecules, IL-8 release, and neutrophil chemotaxis [75]. Endothelial cells have specific receptors for SP and CGRP. These neuropeptides influence endothelial cell functions through their receptors. SP and CGRP have several effects that can play a role in the pathogenesis of vasculitis, via endothelial cells and neutrophils. The aforementioned effects of SP and CGRP on vasodilatation, vascular permeability, and endothelial-associated inflammation suggest that they may have a role in vasculitic events [76]. In conclusion, an increase in serum SP and CGRP levels in BD was found in different studies. This suggests that the neuropeptides may be contributing to BD pathogenesis by affecting vasculitic events.

### 4.6. Natriuretic Peptides in Behçet's Disease

Natriuretic peptides (NPs) maintain an important endocrine-paracrine influence over many vascular parameters. NPs have a fundamental role in cardiovascular remodeling, volume homeostasis, and the response to ischemia. Moreover, the NP level is associated with cardiac problems such as heart failure and thromboembolism [77, 78]. Cardiovascular involvement in BD includes coronary arteritis, coronary artery aneurysm, myocarditis, pericarditis, acute myocardial infarction, silent myocardial ischemia, intracardiac thrombus, heart failure, vasculitis, venous occlusions, arterial aneurysms, and/or arterial occlusions [79]. Yağci et al. studied serum atrial natriuretic peptide (ANP), brain natriuretic peptide (BNP), and C-type natriuretic peptide (CNP) levels in Behçet's patients with active and inactive period. Serum ANP concentrations of the BD patients in the active subgroup were significantly lower than those of the healthy controls. In this study, when compared with healthy controls, serum BNP levels were found to be significantly higher in both the active and inactive BD subgroups. When the study groups were evaluated on the basis of CNP levels, the active BD subgroup had significantly decreased CNP levels in comparison with the inactive and control groups [80]. ANP has been linked to the immune system, and it regulates the balance between TH1 and TH2 responses [81–83]. It may be supposed that decreased CNP levels in the patients may be one of the consequences of the endothelial dysfunction in BD. 

### 4.7. Stress Management in BD

The interaction between the physical and psychological factors seems reasonable in the experience of a BD. Numerous studies have provided a strong basis for considering the role of psychosocial factors on the course the rheumatic disease. Gur et al. established that arthropathy was one of the common manifestations of BD. Arthritis in BD affects considerably patients' pain levels and quality of life [84]. Koçak et al. conclude that depressive mood according to the BDI scale correlates with the sexual status of BD patients, and this may be because of the depressive effect of BD as a chronic disease [85]. Taner et al. observed that patients with BH had more depression and anxiety scores than in psoriatic patients [86]. Uğuz et al. observed that concurrent major depression had a negative impact on quality of life of BD patients and that quality of life was negatively correlated with the severity of depressive symptoms. Behçet's disease was a multisystemic inflammatory disorder associated with high levels of depressive symptoms and lower quality of life [87].

Psychological/psychiatric interventions could usefully be included in the normal Behçet's patients assessment, as it should both improve patient care and be cost effective. Counseling and psychotropic medications can benefit patients with depression or anxiety related to their skin problems, and consultation with a dermatologist and, in some cases, a psychiatrist can be beneficial. The effect of skin diseases is considerable and underappreciated. Physicians applying the biopsychosocial model to skin diseases will be rewarded with improved therapeutic alliances and with grateful patients who experience improved quality of life [88].

No specific pharmacological interventions are as yet available to prevent or treat stress triggered skin disorders in humans. However, based on the wealth of data that has recently accumulated in this field, reasonable pharmacological treatment options are slowly coming into sight. Abrogation of mast-cell activation seems to be a promising approach in this endeavor, but, to date, few if any clinically available molecules can effectively inhibit mast-cell activation. Disodium cromoglycate was shown to inhibit rodent mast cells but was a very weak inhibitor of mast-cell cytokine release [89]. Increasing recent evidence indicates that certain flavonols, such as quercetin, are powerful inhibitors of both prestored and newly synthesized mediators from human mast cells [90]. The combination of such flavonoids with proteoglycans, such as chondroitin sulphate, appears to provide synergistic efficacy by inhibiting both activation and secretion of mast cells [91]. Appropriate CRH-R antagonists, when available, might also provide a unique therapeutic approach in skin conditions precipitated or worsened by stress [92]. Further, the prototypic stress-associated neuropeptide SP may be blocked by the application of a high-affinity neurokinin-1 receptor antagonist [93]. Thus, neurokinin-1 receptor antagonists might be useful in alleviating stress-induced hair loss and skin inflammation. NGF receptor p75 antagonists also deserve systematic exploration as candidate “antistress” drugs in the treatment of stress-triggered or stress-aggravated skin disorders, such as psoriasis and stress-induced telogen effluvium [94]. However, one evidently must be very cautious in translating results from murine models to humans. No specific pharmacological intervention—other than antidepressants and anxiolytics—is currently clinically available to manage selectively the impact of psychological stress on skin disorders in humans. However, reasonable pharmacological treatment options are coming into sight. Mast cells could be prominent targets of CRH and related peptides, contributing to neurogenic inflammation; it is therefore reasonable to propose the use of CRHR antagonists. CRHR antagonists (e.g., antalarmin or astressin) would be one class of molecules that could be tested by local administration in the model systems described, especially because higher CRHR-1 gene expression was documented in contact dermatitis [94]. Folates supplementation may be useful for BD patients with hyperhomocysteinemia. And also nonenzymatic antioxidants such as vitamins A, C, E, and carotene, trace elements including selenium, plasma iron, manganese, and zinc might be useful in patients with BD, if they are low [78] (see Table 1).

## 5. Conclusion

However, an adaptive immune system is also crucial in BD, with possibly both external (streptococcal, superantigens) and internal (heat-shock- or organ-specific proteins) antigens driving the pathogenic tissue T-cell infiltrations. Better characterisation of pathogenic immune cell subsets, systemic and local antigens, and abnormal cell-activation mechanisms may help in the future to develop more specific and less toxic immunotherapeutic approaches to the still unsatisfactorily treated BD. There is some clinical evidence suggesting that hormonal alterations can influence the course and disease activity of BD. Behçet's experts should become far more attentive to the effect of psychological stress on skin disorders, not only for the benefit of their patients but also because the skin serves as a very clinically relevant model system for exploring the neuroimmunology of peripheral and central stress responses.

## Figures and Tables

**Table 1:** Key stress protagonists in the BD.

Stress mediators, hormones, and cells	Main biological effect	In BD
Hormones of the HPA axis (CRH, ACTH, glucocorticoids)	Activate mast cells Upregulate production of IL-4, IL-6, IL-10, and IL-13 Inhibit the production of IL-12, IFN-*γ*, and TNF-*α* by antigen-presenting cells and T-helper 1 cells	Partial HPA axis dysfunction [10].

Prolactin	Participates in early and late T-cell activating events; contributes to a proinflammatory and apoptosis-prone environment	Increased, decreased, or normal [26–29].

Sex hormones	Immune modulation, affect HPA axis	Activation of neutrophils by testosterone, lower levels of DHEA-S (ocular BD), increased 17-OH-progesteron, testosterone, estradiol, FSH, LH, T3, T4, and normal TSH levels and androgen receptor density in scrotal skin, increased 21-hydroxylase gene mutations [30–32].

Substance P	Induces inflammation Induces lymphocyte proliferation Activates mast cells	Higher levels in active BD, strong immunoreactivity of SP in vasculitic skin lesions [39, 45].

CGRP	Inhibits proliferation and IL-2 release of T lymphocytes under immune challengesActivates mast cells, induces vascular permeability	Increased or decreased levels in BD [45, 52].

NGF, NEP, neuropeptide degrading enzyme	Promotes “crosstalk” between neuronal and immune cellsActs as autocrine and paracrine factor in the development and regulation of immune cellsPromotes monocyte and macrophage migration through vascular endotheliumActivates mast cells	Strong immunoreactivity in vasculitic skin lesions, Decreased NGF levels in inactive BD (Ocular-BD) [39, 52].

Natriuretic peptides	Endocrine-paracrine influence over many vascular parameters including fluid and electrolyte balance, vasodilatation, smooth muscle proliferation, and the reactivity of immune cells.	Lower ANP concentrations in active BD, higher serum BNP levels in BD, decreased CNP levels in active BD [56].

Heat shock proteins	Activates CD4 and *γδ*-T lymphocytes	Antibodies against bacterial or human HSP-60/65 are capable of cross-reacting with retinal antigensExposure to HSPs results in the proliferation of peripheral T lymphocytes of ocular BD patients Lesional skin of BD contains increased numbers of both HSP-60 and TCR *γδ*-T lymphocytes*γδ*-T lymphocyte is present in tissue specimens, whereas HSP-65 expression is abundantly upregulated in epidermal regions of active skin lesions Elevated HSPs upregulate the expression of the MICA locus in BD patients [68, 69].

Oxidative stress	Activate neutrophil function, chemotaxis, and phagocytosis	Excessive superoxide anion production, raised ADA activity, hydrogen peroxide-induced hydroxyl radical, and malondialdehyde productions [71, 75]

Antioxidative defense	Free radical scavenging	Decreased superoxide dismutase, glutathione peroxidase, and catalase levels [71].

Trace elements and vitamins	Cofactors to antioxidant enzymesNonenzymatic antioxidants	Decreased erythrocyte selenium, plasma iron, manganese, and zinc levels, increased plasma copper, erythrocyte zinc, and manganese levels, lower plasma concentrations of vitamins A, C, E, and *β*-carotene [77, 78].

Neutrophils, monocytes, and complements	Produce a number of proinflammatory cytokines, chemotaxis, active oxygen production, and phagocytosisInnate immune system activation	Hyperfunctions of neutrophils, leukocyte adhesion molecules including P and L selectins, Mac-1 and CD4 expression on peripheral leukocytes, increased plasma myeloperoxidase activity, elevated peripheral white blood cell count, activated monocytes, increased neutrophil motility, and circulating proteins including C3, C4, C5 [79].

Nitric oxide	Free oxygen radical	Decreased serum nitrite and nitrate concentrations, and Glu-Asp298 polymorphisms of endothelial NO synthase gene, increased NO concentration [85–90].

Psychological factors	Regulate the immune system at regional, local, and systemic levels	More depression and anxiety scores, affected quality of life [85–88].
